# Epidemiologic and Clinical Features of Children and Adolescents Aged <18 Years with Monkeypox — United States, May 17–September 24, 2022

**DOI:** 10.15585/mmwr.mm7144a4

**Published:** 2022-11-04

**Authors:** Ian Hennessee, Victoria Shelus, Cristin E. McArdle, Maren Wolf, Sabrina Schatzman, Ann Carpenter, Faisal S. Minhaj, Julia K. Petras, Shama Cash-Goldwasser, Meghan Maloney, Lynn Sosa, Sydney A. Jones, Anil T. Mangla, Rachel E. Harold, Jason Beverley, Katharine E. Saunders, Jeremy N. Adams, Danielle R. Stanek, Amanda Feldpausch, Jessica Pavlick, Megan Cahill, Victoria O’Dell, Moon Kim, Jemma Alarcón, Lauren E. Finn, Maura Goss, Monique Duwell, David A. Crum, Thelonious W. Williams, Katrina Hansen, Megan Heddy, Krystle Mallory, Darby McDermott, Mervin Keith Q. Cuadera, Eric Adler, Ellen H. Lee, Amanda Shinall, Carlen Thomas, Erin K. Ricketts, Tammy Koonce, Dana B. Rynk, Kelly Cogswell, Meagan McLafferty, Dana Perella, Catherine Stockdale, BreeAnna Dell, Mellisa Roskosky, Stephen L. White, Kenneth R. Davis, Rania S. Milleron, Skyler Mackey, L. Anna Barringer, Hollianne Bruce, Debra Barrett, Marisa D’Angeli, Anna Kocharian, Rachel Klos, Patrick Dawson, Sascha R. Ellington, Oren Mayer, Shana Godfred-Cato, Sarah M. Labuda, David W. McCormick, Andrea M. McCollum, Agam K. Rao, Johanna S. Salzer, Anne Kimball, Jeremy A. W. Gold, California Department of Public Health Monkeypox Pediatric Working Group, CDC Monkeypox Pediatric Working Group, California Department of Public Health Monkeypox Pediatric Working Group, Rick Berumen, Giorgio Cosentino, Shiffen Getabecha, Carol Glaser, Kaitlin Grosgebauer, Kathleen Harriman, Monica Haw, Amanda Kamali, Chantha Kath, Elissa H. Kim, Linda S. Lewis, Darpun Sachdev, Maria Salas, Cameron Stainken, Debra A. Wadford, Philip J. Peters, Akanksha Vaidya, CDC Monkeypox Pediatric Working Group, Susan Hocevar Adkins, ; Nicolle Baird, Lisa C. Barrios, Amy Beeson, Dawn Blackburn, Brian F. Borah, Eleanor Click, Whitni Davidson, Romeo R. Galang, Kaitlin Hufstetler, Helena J. Hutchins, Athena P. Kourtis, Maureen J. Miller, Sapna Bamrah Morris, Emily O’Malley Olsen, Nicole M. Roth, Emily Sims, Kevin Chatham-Stephens

**Affiliations:** ^1^CDC Monkeypox Emergency Response Team; ^2^Epidemic Intelligence Service, CDC; ^3^Laboratory Leadership Service, CDC; ^4^Connecticut Department of Public Health; ^5^Division of State and Local Readiness, Center for Preparedness and Response, CDC; ^6^District of Columbia Department of Health, Washington, DC; ^7^Florida Department of Health; ^8^Georgia Department of Public Health; ^9^Idaho Department of Health and Welfare; ^10^Central District Health, Boise, Idaho; ^11^Los Angeles County Department of Public Health, Los Angeles, California; ^12^Maine Center for Disease Control and Prevention, Augusta, Maine; ^13^Maryland Department of Health; ^14^New Hampshire Division of Public Health Services; ^15^New Jersey Department of Health; ^16^New York City Department of Health and Mental Hygiene, New York, New York; ^17^North Carolina Department of Health and Human Services; ^18^Guilford Department of Health and Human Services; ^19^Mecklenburg County Public Health, Charlotte, North Carolina; ^20^Oregon Health Authority; ^21^Philadelphia Department of Public Health, Philadelphia, Pennsylvania; ^22^Public Health – Seattle & King County, Seattle, Washington; ^23^Texas Department of State Health Services; ^24^Alexandria City Health Department, Alexandria, Virginia; ^25^Virginia Beach Department of Public Health, Virginia Beach, Virginia; ^26^Snohomish Health District, Everett, Washington; ^27^Grays Harbor County Public Health, Aberdeen, Washington; ^28^Washington State Department of Health; ^29^Wisconsin Department of Health Services.; California Department of Public Health; California Department of Public Health; California Department of Public Health; California Department of Public Health; California Department of Public Health; California Department of Public Health; California Department of Public Health; California Department of Public Health; California Department of Public Health; California Department of Public Health; California Department of Public Health; California Department of Public Health; California Department of Public Health; California Department of Public Health; California Department of Public Health; California Department of Public Health and CDC; California Department of Public Health and University of California San Francisco.; CDC; CDC; CDC; CDC; CDC; CDC; CDC; CDC; CDC; CDC; CDC; CDC; CDC; CDC; CDC; CDC; CDC; CDC.

Data on monkeypox in children and adolescents aged <18 years are limited (*1*,*2*). During May 17–September 24, 2022, a total of 25,038 monkeypox cases were reported in the United States,^†^ primarily among adult gay, bisexual, and other men who have sex with men (*3*). During this period, CDC and U.S. jurisdictional health departments identified *Monkeypox virus* (MPXV) infections in 83 persons aged <18 years, accounting for 0.3% of reported cases. Among 28 children aged 0–12 years with monkeypox, 64% were boys, and most had direct skin-to-skin contact with an adult with monkeypox who was caring for the child in a household setting. Among 55 adolescents aged 13–17 years, most were male (89%), and male-to-male sexual contact was the most common presumed exposure route (66%). Most children and adolescents with monkeypox were non-Hispanic Black or African American (Black) (47%) or Hispanic or Latino (Hispanic) (35%). Most (89%) were not hospitalized, none received intensive care unit (ICU)–level care, and none died. Monkeypox in children and adolescents remains rare in the United States. Ensuring equitable access to monkeypox vaccination, testing, and treatment is a critical public health priority. Vaccination for adolescents with risk factors and provision of prevention information for persons with monkeypox caring for children might prevent additional infections.

During May 17–September 24, 2022, children and adolescents who received a positive polymerase chain reaction (PCR) test result for MPXV, nonvariola *Orthopoxvirus* (NVO), or generic *Orthopoxvirus* (OPXV) were identified through national surveillance or during CDC clinical consultations. Demographic and exposure characteristics and clinical features of children and adolescents aged <18 years with monkeypox-compatible symptoms^§^ who received a positive NVO, OPXV, or MPXV PCR test result were analyzed. In cases for which PCR test cycle threshold (Ct) results were available, persons whose specimens had NVO, OPXV, or MPXV PCR Ct values ≥34 (potentially indicating a false positive test result) and who had atypical clinical features or no known epidemiologic risk factors^¶^ were excluded.

Data collected included age; sex; gender identity (among adolescents); race and ethnicity; exposure setting and risk behaviors; monkeypox symptoms and lesion distribution; receipt of JYNNEOS vaccine postexposure prophylaxis, tecovirimat (Tpoxx; SIGA Technologies), topical trifluridine (Viroptic; Pfizer Inc.), or vaccinia immune globulin intravenous (VIGIV; Cangene Corporation)**; and hospitalization status. Data were stratified by age group (0–4, 5–12, and 13–17 years). This activity was reviewed by CDC and was conducted consistent with applicable federal law and CDC policy.^††^

During May 17–September 24, 2022, 83 MPXV infections were identified among children and adolescents aged <18 years, including 16 (19%) in children aged 0–4 years, 12 (14%) in children aged 5–12 years, and 55 (66%) in adolescents^§§^ (Table 1). Among 28 children aged 0–12 years, 18 (64%) were boys, and 10 (36%) were girls. Most adolescents were male (48; 89%), six (11%) were female, and information on sex was missing for one. Overall, 38 (47%) children and adolescents were Black, 28 (35%) were Hispanic, 10 (12%) were non-Hispanic White, and five (6%) were of another race and ethnicity; data on race and ethnicity were missing for two.

**TABLE 1:** Demographic and epidemiologic features of children and adolescents aged <18 years with monkeypox — United States, May 17–September 24, 2022

Characteristic*	No. (%) by age group, yrs			
	All (N = 83)	0–4 (n = 16)	5–12 (n = 12)	13–17 (n = 55)
**Sex^†^**				
Male	66 (80)	12 (75)	6 (50)	48 (89)
Female	16 (20)	4 (25)	6 (50)	6 (11)
Unknown, no.	1	0	0	1
**Race or ethnicity**				
Black, non-Hispanic or Latino	38 (47)	7 (44)	5 (42)	26 (49)
Hispanic or Latino	28 (35)	5 (31)	5 (42)	18 (34)
White, non-Hispanic or Latino	10 (12)	3 (19)	2 (17)	5 (9)
Asian, non-Hispanic or Latino	2 (2)	0 (—)	0 (—)	2 (4)
American Indian or Alaska Native, non-Hispanic or Latino	1 (1)	0 (—)	0 (—)	1 (2)
Native Hawaiian or other Pacific Islander, non-Hispanic or Latino	1 (1)	1 (6)	0 (—)	0 (—)
Other, non-Hispanic or Latino	1 (1)	0 (—)	0 (—)	1 (2)
Unknown, no.	2	0	0	2
**Exposure setting and route**				
Sexual contact^§^	34 (62)	0 (—)	0 (—)	34 (97)
Household contact^¶^	19 (35)	13 (93)	6 (100)	0 (—)
Other**	2 (4)	1 (7)	0 (—)	1 (3)
Unknown, no.	28	2	6	20

* Percentages were calculated using nonmissing data.

^†^ Reported as sex assigned at birth. Gender identity was known for 25 (45%) adolescents. One adolescent whose assigned sex at birth was female identified as a transgender male.

^§^ For these persons, all of whom were aged ≥15 years, direct skin-to-skin sexual contact was the presumed mode of spread. Among 48 male adolescents, 23 (48%) reported male-to-male sexual contact, four (8%) reported sexual contact with a female, and five (10%) reported sexual contact with a person whose sex was not specified. One female adolescent reported recent sexual encounters with a male, but further details were unavailable; another adolescent who identified as a transgender person or teen reported recent sexual contact with a male adolescent.

^¶^ In 17 cases among children aged 0–12 years, direct skin-to-skin contact occurred between the child and an adult with monkeypox who was caring for the child in the setting of routine caregiving activities. In one instance, no direct skin-to-skin contact was noted, but the child shared a living space with the index patient, likely with frequent contact with shared materials (e.g., towels). In the remaining instance, further details about the exposure were unavailable.

** In one instance, direct skin-to-skin contact occurred with an adult with monkeypox who held the child, but the exposure occurred outside the household setting. In another instance, an adolescent shared a bed with another adolescent who had a rash, but further details were unavailable.

Among 20 (71%) children aged 0–12 years with available exposure data, 19 were exposed in the household setting; for 17 of these children, the reported exposure was direct skin-to-skin contact that routinely occurs between a child and an adult caregiver. In another instance, fomite transmission (e.g., towels shared with a caregiver with monkeypox) was the suspected route of exposure because the index patient and the child had shared a living space without direct skin-to-skin contact. In the remaining instance, further information about the exposure was unavailable. One nonhousehold exposure occurred when an adult with monkeypox held a child outside the household setting. In two instances, adult caregivers contracted monkeypox after caring for children with monkeypox in household settings; the suspected exposure routes were skin-to-skin contact during diapering and other routine child care activities.

Among 35 (64%) adolescents with available exposure data, 32 were males with direct skin-to-skin sexual contact as the presumed mode of spread: 23 (72%) reported male-to-male sexual contact, four (13%) reported male-to-female sexual contact, and five (16%) reported sexual contact with a person whose sex was not specified. One female adolescent reported recent sexual contact with a male adolescent, but further details were unavailable; another adolescent who identified as a transgender male reported recent sexual contact with a male adolescent. One female adolescent had shared a bed with another adolescent who had a rash, but further details were unavailable.

Among the 28 children aged 0–12 years with monkeypox, lesions most commonly occurred on the trunk; no child had anogenital lesions; 10 (36%) received tecovirimat, one (4%) received VIGIV, and three (11%) received topical trifluridine (Table 2). Two children aged 0–4 years were hospitalized with diffuse rash and eyelid involvement; both recovered without complications and were discharged.^¶¶^ One child aged 5–12 years was hospitalized for periorbital cellulitis and conjunctivitis; this child received oral tecovirimat and topical trifluridine and recovered.

**TABLE 2:** Clinical features and treatment of children and adolescents aged <18 years with monkeypox — United States, May 17–September 24, 2022

Characteristic*	No. (%) by age group, yrs			
	All (N = 83)	0–4 (n = 16)	5–12 (n = 12)	13–17 (n = 55)
**Condition**				
Immunocompromise^†^	2 (2)	0 (—)	0 (—)	2 (4)
Atopic dermatitis or other exfoliative condition	6 (7)	3 (19)	1 (8)	2 (4)
**Symptom**				
Rash^§^	83 (100)	16 (100)	12 (100)	55 (100)
Fever	29 (35)	4 (25)	3 (25)	22 (40)
Malaise	30 (36)	4 (25)	3 (25)	23 (42)
Lymphadenopathy	24 (29)	3 (19)	2 (17)	19 (35)
**Location of lesion** ^¶^				
Head, face, mouth, or eyes**	34 (41)	7 (44)	3 (25)	24 (44)
Trunk	46 (55)	9 (56)	4 (33)	33 (60)
Extremities	26 (31)	5 (31)	4 (33)	17 (31)
Genitals or perianal area	33 (40)	0 (—)	0 (—)	33 (60)
**No. of lesions**				
<5	6 (19)	4 (44)	1 (25)	1 (5)
5–10	12 (38)	2 (22)	1 (25)	9 (47)
11–20	9 (28)	2 (22)	2 (50)	5 (26)
>20	5 (16)	1 (11)	0 (—)	4 (21)
Unknown	51	7	8	36
**Treatment administered**				
Tecovirimat^††^	18 (22)	8 (50)	2 (17)	8 (15)
Vaccinia immune globulin intravenous	1 (1)	1 (6)	0 (—)	0 (—)
JYNNEOS^§§^	2 (2)	0 (—)	0 (—)	2 (4)
**Outcomes**				
Hospitalization	9 (11)	2 (13)	1 (8)	6 (11)
Death	0 (—)	0 (—)	0 (—)	0 (—)

* Percentages calculated using nonmissing data.

^†^ Two adolescents had recently received a diagnosis of HIV infection; one received this diagnosis while hospitalized with monkeypox, and the other received the diagnosis in an outpatient setting.

^§^ Rash was part of the case definition and is typically required for monkeypox testing.

^¶^ Lesions could occur on more than one body site.

** Included two children aged 0–4 years who had eyelid involvement and received topical trifluridine as potential prophylaxis for ocular monkeypox and one child aged 5–12 years who received topical trifluridine for conjunctivitis.

^††^ Eighteen persons received oral tecovirimat, including six of the nine persons who were hospitalized; one hospitalized adolescent aged 13–17 years initially received intravenous tecovirimat before being switched to oral tecovirimat.

^§§^ One adolescent received JYNNEOS as postexposure prophylaxis 6 days before the onset of monkeypox symptoms; the timing of JYNNEOS receipt was unknown for the other adolescent.

Among the 55 adolescents, lesions most commonly occurred on the trunk (33, 60%) and the genitals or perianal area (33, 60%). Eight (15%) received tecovirimat. Six (11%) adolescent patients were hospitalized. For five adolescent patients, reasons for hospitalization included pain management, treatment of secondary bacterial infections, and systemic symptoms with rash; three of these adolescents received oral tecovirimat, and whether the other two received tecovirimat is unknown; one adolescent received a new diagnosis of HIV infection during hospitalization. Another adolescent was hospitalized to ensure adequate isolation but had mild symptoms and did not receive monkeypox-directed therapies. All adolescents were discharged and recovered.

Overall, no children or adolescents received ICU-level care or died. No reported case during the investigation timeframe was known to be associated with sexual abuse.

Ten distinct instances were investigated in which a child or adolescent with monkeypox attended a child care facility (two) or school (eight) while symptomatic; no instance of secondary transmission in these settings was identified. JYNNEOS vaccination was offered to close contacts in at least four situations, and in one instance more than 15 other students and staff members received JYNNEOS postexposure prophylaxis.

## Discussion

MPXV infections in children and adolescents during May 17–September 24, 2022, constituted a small percentage (0.3%) of total U.S. monkeypox cases, and no children or adolescents with monkeypox received ICU-level care or died. However, consistent with disparities observed during the ongoing monkeypox epidemic (*3*), which are likely related to longstanding inequities in the social determinants of health,*** monkeypox in children and adolescents occurred disproportionately among Black and Hispanic children and adolescents compared with U.S. race and ethnicity percentage distributions of persons aged <18 years.^†††^ This finding underscores the continued need for public health efforts to ensure equitable access to monkeypox vaccination, testing, treatment, and information about prevention measures. Similar to findings reported from Spain (*1*), exposure characteristics differed between younger children and adolescents: younger children most often acquired infection after direct skin-to-skin contact with a caregiver or household member known to have monkeypox, whereas exposure characteristics among adolescents were similar to those most commonly reported among adults (i.e., sexual contact) (*3*). Adults with monkeypox who interact with children in the household setting should follow transmission prevention guidelines, which outline measures to prevent the spread of monkeypox in households (*4*), and caregivers who are symptomatic and believe they might have been exposed should try to limit skin-to-skin contact with children, including by covering lesions. In addition, health care providers caring for sexually active adolescents, particularly males who have male-to-male sexual contact, should consider offering vaccination, should provide education on prevention of monkeypox, and should provide testing for HIV and other STIs (*5*).

Limited data, based on infections involving Clade I MPXV rather than the Clade IIb virus causing the current epidemic, suggested that children aged <8 years might be at higher risk for severe disease than are older persons (*6*,*7*). However, the clinical signs and symptoms reported in children and adolescents in this report were broadly similar to findings from Spain and U.S. national surveillance data for cases overall (*1*,*3*), with most children experiencing a mild-to-moderate clinical course. Clinicians caring for children and adolescents should be aware of available clinical guidance for the diagnosis and treatment of monkeypox^§§§^ and of the potential for severe disease, particularly in persons with profound immunocompromise (e.g., those with advanced HIV disease or undergoing chemotherapy for cancer) (*8*).

No secondary transmission was identified during instances when children attended school or a child care facility while symptomatic, although incomplete case ascertainment and reporting might have limited detection of such events. The absence of known secondary transmission in schools and child care facilities despite the presence of symptomatic persons in these settings suggests that widespread child-to-child transmission might be unlikely.^¶¶¶^ Regardless of age, contacts of persons with monkeypox should be monitored, and JYNNEOS vaccination postexposure prophylaxis should be considered based on an exposure risk assessment and individual risk for severe disease (*7*,*9*).

The findings in this report are subject to at least three limitations. First, data regarding exposure characteristics were missing for one third (34%) of children and adolescents aged <18 years, potentially because of difficulty reaching caregivers or adolescents for interviews or interviewee reluctance to disclose potentially sensitive information because of fear of stigma. Second, exposure misclassification might have occurred because of recall or social desirability bias. Finally, this report could potentially underestimate the number of MPXV infections occurring if children and adolescents aged <18 years with monkeypox did not receive testing. Nonetheless, caution is needed when ordering monkeypox tests and interpreting laboratory results for persons with low pretest probability of infection, because false positive test results can lead to unnecessary or inappropriate medical treatment (*10*).

This analysis found that monkeypox in children and adolescents aged <18 years has been rare during the current outbreak and most infections were not severe. Public health messaging should emphasize transmission prevention guidelines for persons with monkeypox who interact with newborns, infants, and children in household settings (*4*,*9*). In addition, health care providers caring for sexually active adolescents, particularly male adolescents who have male-to-male sexual contact, should encourage vaccination for eligible persons and should provide testing for HIV and other sexually transmitted diseases.

**Summary**

**What is already known about this topic?**

Data on epidemiologic and clinical characteristics of monkeypox in persons aged ≤12 years (children) and adolescents during the ongoing 2022 monkeypox outbreak are limited.

**What is added by this report?**

During May 17–September 24, 2022, *Monkeypox virus* (MPXV) infections in children and adolescents aged <18 years were rare, representing 0.3% of all U.S. cases; none resulted in critical illness or death. Younger children typically acquired MPXV infection after skin-to-skin contact with a household member with monkeypox during caregiving activities; adolescents were most frequently exposed through male-to-male sexual contact.

**What are the implications for public health practice?**

Additional monkeypox cases in children and adolescents might be prevented through strengthened vaccination efforts and education around preventive measures and sexual health.

## Footnotes

† https://www.cdc.gov/poxvirus/monkeypox/response/2022/us-map.html (Accessed October 4, 2022).

§
https://www.cdc.gov/poxvirus/monkeypox/symptoms.html

¶
https://www.cdc.gov/poxvirus/monkeypox/clinicians/case-definition.html

** https://www.fda.gov/news-events/press-announcements/monkeypox-update-fda-authorizes-emergency-use-jynneos-vaccine-increase-vaccine-supply; https://www.cdc.gov/poxvirus/monkeypox/clinicians/Tecovirimat.html; https://www.fda.gov/media/78174/download; https://www.cdc.gov/poxvirus/monkeypox/clinicians/ocular-infection.html

†† 45 C.F.R. part 46.102(l)(2), 21 C.F.R. part 56; 42 U.S.C. Sect. 241(d); 5 U.S.C. Sect. 552a; 44 U.S.C. Sect. 3501 et seq.

§§ During the investigation period, CDC received notifications of 109 children and adolescents aged <18 years who received a positive PCR test result for MPXV, NVO, or OPXV, among whom 26 cases were ruled out after further investigation based on high Ct values on NVO, OPXV, or MPXV PCR testing, negative repeat testing, or absence of epidemiological risk factors.

¶¶ These children were aged <1 year. Both received oral tecovirimat, and both also received topical trifluridine as potential prophylaxis for ocular monkeypox. One received VIGIV because of their very young age (infant), their immature immune system, and certain other factors.

***
https://www.cdc.gov/socialdeterminants/about.html

†††
https://www.childstats.gov/americaschildren/glance.asp

§§§
https://www.cdc.gov/poxvirus/monkeypox/clinicians/treatment.html

¶¶¶
https://www.cdc.gov/poxvirus/monkeypox/community/school-faq.html

## References

[1] Aguilera-Alonso D, Alonso-Cadenas JA, Roguera-Sopena M, Lorusso N, Miguel LGS, Calvo C. Monkeypox virus infections in children in Spain during the first months of the 2022 outbreak. Lancet Child Adolesc Health 2022;6:e22–3. 10.1016/S2352-4642(22)00250-4 36058226 PMC9555952

[2] Saunders KE, Van Horn AN, Medlin HK, Monkeypox in a young infant—Florida, 2022. MMWR Morb Mortal Wkly Rep 2022;71:1220–1. 10.15585/mmwr.mm7138e3 36136958 PMC9531566

[3] Philpott D, Hughes CM, Alroy KA, ; CDC Multinational Monkeypox Response Team. Epidemiologic and clinical characteristics of monkeypox cases—United States, May 17–July 22, 2022. MMWR Morb Mortal Wkly Rep 2022;71:1018–22. 10.15585/mmwr.mm7132e3 35951487 PMC9400536

[4] CDC. Monkeypox: isolation and infection control at home. Atlanta, GA: US Department of Health and Human Services, CDC; 2022. Accessed September 15, 2022. https://www.cdc.gov/poxvirus/monkeypox/clinicians/infection-control-home.html

[5] CDC. Monkeypox: safer sex, social gatherings, and monkeypox. Atlanta, GA: US Department of Health and Human Services, CDC; 2022 Accessed September 26, 2022. https://www.cdc.gov/poxvirus/monkeypox/prevention/sexual-health.html

[6] Huhn GD, Bauer AM, Yorita K, Clinical characteristics of human monkeypox, and risk factors for severe disease. Clin Infect Dis 2005;41:1742–51. 10.1086/498115 16288398

[7] CDC. Monkeypox: clinical considerations for monkeypox in children and adolescents. Atlanta, GA: US Department of Health and Human Services, CDC; 2022. Accessed September 15, 2022. https://www.cdc.gov/poxvirus/monkeypox/clinicians/pediatric.html

[8] Miller MJ, Cash-Goldwasser S, Marx GE, Severe monkeypox in hospitalized patients—United States, August 10–October 10, 2022. MMWR Morb Mortal Wkly Rep 2022;71:1412–7. 10.15585/mmwr.mm7144e1 36327164 PMC9639440

[9] CDC. Monitoring and risk assessment for persons exposed in the community. Atlanta, GA: US Department of Health and Human Services, CDC; 2022. Accessed September 15, 2022. https://www.cdc.gov/poxvirus/monkeypox/clinicians/monitoring.html

[10] Minhaj FS, Petras JK, Brown JA, ; CDC Monkeypox Emergency Response Team. *Orthopoxvirus* testing challenges for persons in populations at low risk or without known epidemiologic link to monkeypox—United States, 2022. MMWR Morb Mortal Wkly Rep 2022;71:1155–8. 10.15585/mmwr.mm7136e1 36074752 PMC9470221

